# Blood Metabolites and Feed Utilization Efficiency in Thai-Native-Anglo-Nubian Goats Fed a Concentrate Diet Including Yeast Fermented Palm Kernel Cake Instead of Soybean Meal

**DOI:** 10.3390/vetsci9050235

**Published:** 2022-05-12

**Authors:** Pin Chanjula, Chanadol Supapong, Puwadon Hamchara, Anusorn Cherdthong

**Affiliations:** 1 Animal Production Innovation and Management Division, Faculty of Natural Resources, Hat Yai Campus, Prince of Songkla University, Songkhla 90112, Thailand; 2 Department of Animal Science, Faculty of Agriculture, Rajamangala University of Technology Srivijaya, Nakhon Si Thammarat 80240, Thailand; chanadol.s@rmutsv.ac.th; 3 Program of Animal Science, Faculty of Science and Technology, Suratthani Rajabhat University, Suratthani 84100, Thailand; puwadon.ham@sru.ac.th; 4 Tropical Feed Resource Research and Development Center (TROFREC), Department of Animal Science, Faculty of Agriculture, Khon Kaen University, Khon Kaen 40002, Thailand; anusornc@kku.ac.th

**Keywords:** yeast, palm kernel cake protein, nutrient utilization, rumen fermentation, goats

## Abstract

Feed is the most expensive component in goat production. Hence, lowering it is crucial to increasing producer profitability. The microbial community in rumen is vital for nutritional digestion and absorption in ruminants. Live yeast and yeast-based products generated from the strain *Saccharomyces cerevisiae* (commercial strain) are actively being used and investigated. The purpose of this study was to investigate the effects of substituting soybean meal (SBM) in concentrate diets with yeast-fermented palm kernel cake protein (YFPKCP) on dry matter intake, digestibility, blood markers, and nitrogen balance. Five crossbred Thai Native-Anglo-Nubian goats (50% Thai Native goats with 50% Anglo-Nubian goats) weighing an average of 27 ± 2 kg were randomly allocated to one of five diets using a 5 × 5 Latin square design: 0, 25, 50, 75, and 100% YFPKCP replacement for SBM. Plicatulum hay (*Paspalum plicatulum* Michx.) was provided ad libitum. There were no significant differences in dry matter (DM) intake among treatments, but the apparent digestibility of DM, crude protein (CP), neutral detergent fiber (NDF), and acid detergent fiber (ADF) were affected (*p* < 0.05) by including YFPKCP in diets. They also tended to be slightly lower for goats fed the diet containing 100% YFPKCP replacement for SBM compared to other treatments. Ruminal pH, ammonia-nitrogen (NH_3_-N), blood glucose, and packed cell volume were equivalent among treatments. On the other hand, replacement YFPKCP reduced digestibility and N absorption by up to 75% (*p* < 0.05). Furthermore, there was no difference in total volatile fatty-acid concentration among goats fed YFPKCP as a substitute for SBM. According to the results of this study, the level of YFPKCP in the concentrate replacement of SBM for goats fed plicatulum hay should be 75%.

## 1. Introduction

The meat-goat market has lately grown in popularity [1], presenting a host of new opportunities for diversifying farm earnings. However, because feed is the most expensive component of goat production, lowering it is crucial to enhancing producer earnings. Feeds that are both cost-effective and easy to handle will soon be required for meat goat producers. Oil palm (*Elaeis guineensis* Jacq.) is widely accessible and belongs to a well-developed oil-producing industry [2]. Palm kernel cake (PKC) is a by-product of palm oil production that is abundant in Southeast Asia, Indonesia, Malaysia, and the southern part of Thailand. Palm kernel cake, also known as palm kernel meal, has demonstrated to be an excellent feed element for a variety of ruminants and is widely accessible in tropical countries [3,4]. PKC has ametabolizable energy (ME) content of 20.6 MJ/kg [5]. The nutritional value of forages for ruminant feeding has been tested utilizing the procedure of protein enrichment of animal feed employing microorganisms in a semi-solid culture. Punj [6] discovered that, depending on the method used to extract the oil, palm kernel meal contains 12–23% DM of crude protein and has an in vitro dry matter digestibility of 70–80% [7]. However, because of its high neutral detergent fiber (NDF) content (60–70% NDF) and low palatability, PKC’s application is limited [5,6]. In ruminant nutrition, introducing microbial fermentations to the feed, such as a *Saccharomyces cerevisiae* culture, has become a regular practice and has caused beneficial changes in the activity and numbers of rumen microbes. *S. cerevisiae* fermentation products were proven to be especially beneficial during times of stress and disease, lowering rumen pH oscillations and enhancing dry matter intake [8]. This might be related to alterations in the rumen microbial community which would result in changes in ruminal VFA production [9].

Furthermore, the effect of *S. cerevisiae* fermentation products on dairy cows can enhance milk production and weight gain in growing cattle [10]. When yeast was added to dairy lactating cows, it enhanced milk quality [11], altered feed intake [12], and improved immunological function [13]. Cellulolytic and lactate-utilizing bacteria in the rumen stimulation increased fiber digestion, and greater microbial protein flow from the rumen are all frequent production responses attributed to yeast that may be advantageous for feedlot cattle fed high-grain diets [14]. The inclusion of yeast culture in animal nutrition has a positive effect that improves non-starch polysaccharide degradation which can increase the energy concentration and the release of nutrients [15]. Ruminant feeding of *S. cerevisiae* products can modify the rumen environment, increasing populations of microorganisms associated with fiber digestion [16], lactic acid utilization, and ruminal pH [17]. This may contribute to reducing the cost of production in ruminant systems using low-quality animal feeding by-products as major components, replacing the much more expensive cereal grain and protein source. However, the application of yeast fermented palm kernel cake (YFPKCP) as a SMB substitute has not yet been evaluated. The aim of this experiment was to examine how adding YFPKCP in replacement of SBM in a concentrate diet affected feed utilization and rumen fermentation characteristics, volatile fatty acid profiles, and the nitrogen balance of Thai-native-Anglo-Nubian goats.

## 2. Materials and Methods

### 2.1. Dietary Preparation of Yeast Fermented Palm Kernel Cake (YFPKCP)

To simulate yeast, 20 g of *S. cerevisiae* (Berry Yuker Specialities Company Limited, Bangkok, Thailand) was mixed with 100 mL of water and 20 g of sugar and kept at room temperature (25 ± 2 °C) for 2 h with a pH of 4.5–5.0 before being flushed with oxygen for 18 h. *S. cerevisiae* was mixed with agar medium and molasses: urea: water at a ratio of 24:72:100 (a culture medium: yeast inoculation), and incubated for 60 h at room temperature ([18,19]). The palm kernel cake was obtained in the south of Thailand and inoculated with *S. cerevisiae* before being ensiled in a plastic, sealed container under anaerobic conditions. The samples were sun-dried for three days (25–32 °C) before being analyzed for their component and chemical content (Table 1).

### 2.2. Animals, Experimental Design, and Feeding

The study was conducted under approval record No. 48/2017 of the Animal Ethics and Care issued by Prince of Songkla University. The goats used in this experiment were obtained from the Animal Farm Department, Faculty of Natural Resources, Prince of Songkla University. Because the Animal Farm Department has a limited number of animals with the same condition (breed, BW, age, etc.), it is difficult to offer 10 animals for the current study. Hence, only a few animals were employed. The study, on the other hand, was carried out with caution and control in order to avoid any unintentional mistakes produced by humans, animals, or the environment. Five male crossbreed Thai native-Anglo-Nubian goats (50% Thai Native goats with 50% Anglo-Nubian goats) with an average live weight of 27 ± 2 kg were randomly assigned to one of five meals (0, 25, 50, 75, and 100% YFPKCP substitution for soybean meal (SBM)), respectively, using a 5 × 5 Latin square design. Each goat was housed in a metabolism cage with access to water and mineral blocks at all times. During each period, all goats were fed a 2% BW (DM as fed) concentrate diet and plicatulum hay (PH, *Paspalum plicatulum*, Michx.) was provided ad libitum, with 10% PH refusals allowed (Table 2). The goats were fed twice daily at 8:00 a.m. and 4:00 p.m. Each experimental period lasted for 21 days. The first 15 days were utilized to adjust to the new treatment and monitor feed intake, while the final 6 days were used to determine digestibility using the total collection method [20]. This included 5 days of total feces and urine collection, and the final day for collection of rumen fluid and blood.

### 2.3. Sample Collection and Sampling Procedures

During the last 6 days of each period of the digestibility trial, rumen fluid was taken from all animals using a stomach tube (0.3 cm internal diameter with a length of 1.5 m) at 0 and 4 h post-feeding to verify the effect of rumen fermentation after feeding, and for examination of pH, NH_3_-N, rumen microbe, and volatile fatty acid (VFA) content at the end of each period. The pH of rumen fluid was determined using a portable pH and temperature meter (Hanna Instruments HI 8424 microcomputer, Singapore). The NH_3_-N evaluation was made using 5 mL of sulphuric acid (H_2_SO_4_) mixed with 45 mL of rumen fluid. The solution was centrifuged at 16,000× *g* for 15 min for NH_3_-N analysis by steam distillation with a Kjeltech Auto 1030 analyzer (Tecator, Hoganiis, Sweden). VFAs analysis was performed using high-performance liquid chromatography [21]. Blood samples were drawn from the jugular vein at 0 and 4 h after feeding and placed in tubes containing 12 mg of EDTA. Plasma was separated by centrifugation at 2500× *g* for 15 min and tested for blood urea nitrogen (BUN) [22], blood glucose, and packed cell volume (PCV) using commercial kits (No. 640, Sigma Chemical Co., St. Louis, MI, USA).

Feeds and fecal samples were collected every period to be chemically analyzed for DM, nitrogen, ash, CP, and acid detergent fiber (ADF), following the method of AOAC [23]. The detergent fiber standards technique was used to estimate the neutral detergent fiber (NDF) in samples. Urine samples were tested for urinary N using the AOAC’s Kjeldahl technique [23], and N utilization was estimated. Rumen fluid was diluted in a solution of 10% formalin in sterilized 0.9% saline to dilution, and the counts of bacteria, protozoa, and fungus were assessed using a haemocytometer and the direct counting microscopic technique (Boeco, Hamburg, Germany).

### 2.4. Analyses Statistical

The statistical analysis accounted for the 5 × 5 Latin square design using the GLM and treatment means, procedures of SAS [24] (SAS Inst. Inc., Cary, NC, USA). The following model was used to analyze the data:

Y_*ijk*_ = µ + *M_i_* + *A_j_* + *P_k_* + *C_l_* + *B_m_ +*
*ε_ijk_*

where: Y_*ijk*_, observation from goat *j*, receiving diet *i*, in period *k*; μ, the overall mean, *M_i_*, the effect of the different treatments (*i* = 1, 2, 3, 4, 5), *A_j_*, the effect of animal (*j* = 1, 2, 3, 4, 5), *P_k_*, the effect of the period (*k* = 1, 2, 3, 4, 5), C*_l_*, the effect of collection time, *B_m_* the effect of interaction between treatments and collection time, and *ε_ijk_* the residual effect.

To find differences between means, the treatment means were statistically analyzed using Duncan’s Multiple Range Test [25], and significance was defined at a *p* value of 0.05 for differences in means and trends. Linear and quadratic effects were used to determine orthogonal polynomials for diet responses.

## 3. Results and Discussion

### 3.1. Feed Utilization Effectiveness

Table 3 shows the effects of varying degrees of SBM substitution with YFPKCP in goats. Roughage and concentrate consumption were comparable between treatments (*p* > 0.05) with the total intake ranging from 20.54 to 27.19 g/kg BW^0.75^. It was found that the level of YFPKCP in the concentrate replacement of SBM had no effect on total DMI in growing goats. The apparent digestibility of DM, OM, CP, NDF, and ADF was lowered when SBM was replaced with 100% YFPKCP. Replacement SBM by YFPKCP at 25–75% had no effect on DM, OM, or CP apparent digestibility when compared to 0 percent (SBM only). Feeding YFPKCP at 75% enhanced NDF and ADF apparent digestibility compared to the other levels. The increase of YFPKCP in the diet was because of the associated high fibrous fraction (ADF and ADL) and EE content. The YFPKCP has a high level of non-starch polysaccharides. The introduction of 100% YFPKCP (5.82% EE) reduced digestibility, particularly for ADF digestion and rumen microbial fermentation [26,27]. Feed intake, digestibility, fermentation process, and bacterial proliferation in the rumen were all affected by the fat content of up to 5% in a diet [28]. Furthermore, the lower digestibility of using 100% YFPKCP instead of SBM may have contributed to a lower true protein in the diet. Saxena et al. [29] found a true protein supplementation to be more effective than NPN supplementation. Similarly, Huntington and Archibeque [30] found that ruminants fed true protein had higher protein digestibility than those fed urea or NPN.

### 3.2. Rumen Fermentation Characteristics

The rumen parameters of temperature, pH, NH_3_-N, and BUN were all measured (Table 4). Overall, ruminal temperature and pH were within the ranges considered to be favorable for microbial fiber and protein digestion [31]. YFPKCP diets had no influence on ruminal NH_3_-N levels, which varied from 14.14 to 16.71 mg/dL. Ruminal NH_3_-N concentrations exceeded 5–8 mg/dL which is the optimal amount for microbial protein production [32]. Nitrogen supplies from protein breakdown in the rumen are combined with free amino acids, peptides, and ammonia from amino acid deamination to produce microbial protein synthesis [33,34]. The use of ammonia in the rumen is inextricably linked to the availability of carbohydrates [35]. The yield of microbial protein synthesis generated in the rumen is maximized when the ratio of available energy or fermentable organic matter to protein or nitrogen is adjusted [36]. Meanwhile, at 4 h post-feeding, the ammonia concentration in the rumen tends to increase due to protein degradation by the rumen bacteria. The NH_3_-N produced in the rumen is either used by the rumen bacteria for maintenance and growth or transformed into urea in the liver and excreted in the urine when the supply exceeds the requirement.

### 3.3. Ruminal Volatile Fatty Acid (VFA) Profiles

The molar ratios of the VFA profile were unaffected by the YFPKCP replacement SBM (Table 5). Total VFA, acetic acid, propionic acid, and butyric acid had mean values of 75.0 to 79.2 mM, 61.7 to 62.6 mM, 26.2 to 27.1 mol/100 mol, and 10.7 to 11.5 mol/100 mol, respectively, demonstrating that fermenting palm kernel cake with yeast may improve quality and make it comparable to SBM [37]. It was postulated that yeasts might help fungi to colonize lignin structures. The breakdown of cellulose filter paper by *Neocallimastix frontalis* was similarly accelerated in the presence of live yeast cells in the same studies. This impact has several routes of action, one of which is the source of thiamine, a nutrient required by rumen fungi for zoosporogenesis. It has also been proven that some yeast strains can increase the proliferation and activity of fibrolytic bacteria.

### 3.4. Rumen Microorganisms

When the amount of YFPKCP replacement SBM was increased, bacteria and fungi levels increased significantly (*p* < 0.05; Table 6). Enhanced ammonia incorporation into microbial protein and increased flow and absorption of amino acids, as well as altered endogenous nitrogen metabolism, improved nitrogen intake consumption in ruminants fed yeast culture. In several experimental meals, adding yeast to the control ration enhanced NH_3_-N levels. Protozoal populations, on the other hand, were not statistically different (*p* > 0.05). Protozoal populations at 0 and 4 h post feeding, as well as total protozoal populations, tended to decrease (*p* = 0.07) in goats fed 25–100% YFPKCP replacement for SBM, compared to goats not fed YFPKCP. This is because fatty acids in oil in the pulp of oil palm kernels are poisonous. According to Galbraith and Miller [38], long-chain fatty acids are more dangerous to microbial cells than short-chain fatty acids. In comparison to other groups, the protozoal population in palm cake kernel treated cattle and sheep tended to decrease [39]. Thus, the inclusion of PKC in diets can reduce the protozoal population, altering the ruminal environment by lowering protozoal numbers while indirectly enhancing bacterial numbers and activity. Moreover, Garcia et al. [40] discovered that supplementing live yeast cells in concentrated feed using corn as the major energy source can lower entodinidae and holotrichidae populations in goats. It is possible that live yeast is attached to the oil palm kernel meal in yeast fermented palm kernel meal.

### 3.5. Glucose Concentration and Red Blood Cell Volume

Because yeast culture enhanced microbial activity and increased nitrogen absorption into microbial protein, the addition of YFPKCP to diets had no effect on BUN concentration (*p* > 0.05). Preston et al. [41] discovered that BUN levels were related to rumen ammonia production and were significantly correlated with edible protein consumption. There was no change in blood glucose levels between the YFPKCPs at 0 and 4 h after feeding (*p* > 0.05). The blood glucose levels in the goats varied from 63.92 to 65.00 mg/dL, with normal values being 50–75 mg/dL [42] (Table 7). Many factors influence blood glucose levels in animals, including physiological state [43], disease [44], and sample time [45,46]. According to Mahardika et al. [47], blood glucose levels rose 3–4 h after a meal. This was because propionic acid, a precursor to glucose production, was highest in the rumen 3 h after feeding. PCV at 0 and 4 h after feeding did not differ significantly across groups (*p* > 0.05), with a range of 28.27–29.15%. Rasedee et al. [48] found that PCV values varied depending on the level of diet given to the animals. The PCV value of dairy cows fed 1.75% BW was higher than that of cows fed 1% BW. Furthermore, PCV values in dairy cows (35.91%), buffaloes (38.37%), and cattle (30.37%) were substantially different (*p* < 0.05) depending on the animal species.

### 3.6. Nitrogen Utilization

Total nitrogen intake was not different in goats fed various YFPKCPs (*p* > 0.05) (Table 8). The concentration diet with no YFPKCP had higher urine nitrogen excretion than the other YFPKCP inclusions (*p* < 0.01). This could be due to the amount of protein that has increased CP digestion [49]. Tamminga [50] found a link between high indigestible protein and rumen undegradable protein (RUP), indicating that low total nitrogen excretion promotes nitrogen retention in the body.

All goat groups had a positive nitrogen balance and nitrogen utilization, probably due to receiving a higher nitrogen intake than required. This is most likely owing to NH_3_-N concentrations in the feed exceeding the suggested values for microbial growth and protein synthesis (5–8 mg/dL; Satter and Slyter, [32] or 3.3–8.5 mg/100 mL; Kang-Meznarich and Broderick, [51]). Animals can be kept alive by eating varying levels of YFPKCP as an energy and protein source. Furthermore, as the animal consumes less nitrogen in its diet, it will increase nitrogen retention and decrease nitrogen excretion in order to maintain nitrogen balance [52,53]. Because animals have systems to maintain nitrogen balance in the body, the kidneys limit urea excretion in the urine, allowing urea to be recirculated into the rumen [54,55,56].

## 4. Conclusions

According to the findings of this study, adding YFPKCP to diets had no influence on feed intake, rumen characteristics, blood glucose levels, or volatile fatty acid profiles when SBM was completely substituted. Replacing YFPKCP, on the other hand, lowers digestibility and N absorption by up to 75%. Furthermore, the recommended YFPKCP replacement SBM in a concentrate diet for goats should be 75%.

## Figures and Tables

**Table 1:** Chemical composition of yeast fermented palm kernel cake, oil palm meal, palm kernel, cake, and yeast powder (% DM basis).

Chemical Composition	YFPKCP	Palm Kernel Cake	Yeast Powder
DM	88.61	93.88	95.90
OM	96.12	95.48	93.44
Ash	3.88	4.52	6.56
CP	41.67	17.32	46.97
EE	5.40	5.02	3.12
NDF	47.98	67.20	NA
ADF	32.50	44.63	NA
Ca	0.38	0.37	NA
P	0.56	0.56	NA

YFPKCP: Palm kernel cake fermentation with yeast, DM: dry matter; OM: organic matter; CP: crude protein; EE: ether extract; NDF: neutral detergent fiber; ADF: acid detergent fiber; Ca: calcium; P: phosphorus; NA: not analyze.

**Table 2:** Chemical composition of the experimental diets and plicatulum hay (% DM basis).

Chemical Composition	YFPKCP Substitution SBM in Concentrate (%)					Plicatulum Hay
	0	25	50	75	100	
DM	85.92	84.17	85.81	84.48	85.67	92.35
Ash	6.45	6.55	6.13	6.43	6.53	8.37
OM	93.55	93.45	93.87	93.57	93.47	91.63
CP	15.56	15.20	15.42	15.36	15.33	3.42
EE	3.96	4.22	4.17	4.74	5.82	0.72
NDF	17.96	20.49	23.51	25.62	27.70	81.38
ADF	5.97	7.03	8.00	8.69	9.44	50.02

YFPKCP: yeast fermented palm cake kernel; SBM: soybean meal; DM: dry matter; OM: organic matter; CP: crude protein; EE: ether extract; NDF: neutral detergent fiber; ADF: acid detergent fiber.

**Table 3:** Feed intake and apparent digestibility of fermented palm kernel cake in goats fed plicatulum hay as roughage.

Attribute	YFPKCP Substitution SBM in Concentrate (%)						Contrast	
	0	25	50	75	100	SEM	L	Q
DMI								
Plicatulum hay, kg/d	0.334	0.281	0.248	0.276	0.286	0.031	NS	NS
%BW	1.18	0.99	0.89	1.01	1.03	0.13	NS	NS
g/kg BW^0.75^	27.19	22.88	20.54	23.12	23.64	2.74	NS	NS
Concentrate, kg/d	0.518	0.517	0.499	0.502	0.513	0.038	NS	NS
%BW	1.83	1.82	1.78	1.83	1.83	0.22	NS	NS
g/kg BW^0.75^	42.25	42.09	40.83	41.88	42.12	0.09	NS	NS
Total DMI, kg/d	0.852	0.798	0.747	0.778	0.799	0.06	NS	NS
DMI, %BW	3.01	2.82	2.67	2.84	2.86	0.20	NS	NS
DMI, g/kg BW^0.75^	69.45	64.98	61.38	65.01	65.76	4.49	NS	NS
OMI, kg/d	0.794	0.747	0.710	0.706	0.742	0.05	NS	NS
CPI, g/d	97	92	86	87	88	0.01	NS	NS
NDFI, kg/d	0.439	0.425	0.417	0.489	0.497	0.03	NS	NS
Apparent digestibility, %								
DM	73.15 ^a^	73.02 ^a^	74.29 ^a^	74.78 ^a^	67.35 ^b^	1.56	0.07	*
OM	74.56 ^a^	74.19 ^a^	75.86 ^a^	76.14 ^a^	69.09 ^b^	1.47	0.08	*
CP	68.52 ^a^	70.18 ^a^	72.71 ^a^	69.63 ^a^	61.01 ^b^	1.97	*	NS
NDF	67.42 ^b^	66.71 ^b^	68.32 ^b^	73.03 ^a^	62.26 ^c^	1.41	NS	*
ADF	59.92 ^ab^	57.22 ^ab^	61.26 ^ab^	63.39 ^a^	53.64 ^b^	2.40	NS	NS

YFPKCP: yeast fermented palm cake kernel; SBM: soybean meal; DMI: dry matter intake, BW^0.75^: metabolic body weight; OMI: organic matter intake, CPI: crude protein intake NDFI: neutral detergent fiber intake; DM: dry matter; OM: organic matter; CP: crude protein; NDF: neutral detergent fiber; ADF: acid detergent fiber; SEM: standard error of the mean (*n* = 5); L: linear; Q: quadratic; NS: non-significant (*p* > 0.05); *: significantly different (*p* < 0.05); ^a, b, c^ Within rows not sharing a common superscript are significantly different (*p* < 0.05).

**Table 4:** Rumen fermentation parameters in goats fed various levels of fermented palm kernel cake.

Attribute	YFPKCP Substitution SBM in Concentrate (%)						Contrast	
	0	25	50	75	100	SEM	L	Q
Temperature, °C								
0 h-post feeding	39.1	39.3	39.4	39.2	39.2	0.33	NS	NS
4 h-post feeding	39.8	39.6	39.2	39.4	39.7	0.23	NS	NS
Mean	39.4	39.4	39.3	39.3	39.4	0.16	NS	NS
Ruminal pH								
0 h-post feeding	6.86	6.84	6.93	6.88	6.87	0.06	NS	NS
4 h-post feeding	6.36	6.22	6.22	6.23	6.31	0.05	NS	NS
Mean	6.61	6.53	6.57	6.55	6.59	0.06	NS	NS
Ammonia-nitrogen, mg/dL								
0 h-post feeding	18.57	17.43	14.86	17.43	16.29	1.18	NS	NS
4 h-post feeding	14.86	16.00	14.00	14.29	12.00	1.48	NS	NS
Mean	16.71	16.71	14.43	15.86	14.14	1.13	NS	NS

YFPKCP: yeast fermented palm cake kernel; SBM: soybean meal; SEM: standard error of the mean (*n* = 5); L: linear; Q: quadratic; NS: non-significant (*p* > 0.05).

**Table 5:** Effects of fermented palm kernel cake on volatile fatty acid (VFA) profiles in goats fed on plicatulum hay as roughage.

Attribute	YFPKCP Substitution SBM in Concentrate (%)						Contrast	
	0	25	50	75	100	SEM	L	Q
Total volatile fatty acid (VFA), mmol/L								
0 h-post feeding	73.0	73.9	69.6	79.2	76.3	4.47	NS	NS
4 h-post feeding	78.0	82.3	80.3	79.2	76.3	4.23	NS	NS
Mean	75.5	78.1	75.0	79.2	76.3	4.58	NS	NS
Molar proportion of VFA, mol/100 mol								
Acetate (C_2_)								
0 h-post feeding	60.2	60.6	61.1	62.1	60.4	0.44	NS	NS
4 h-post feeding	64.3	64	63.4	63.1	63.0	1.57	NS	NS
Mean	62.3	62.3	62.3	62.6	61.7	0.84	NS	NS
Propionate (C_3_)								
0 h-post feeding	29.5	29.8	28.5	28	29.3	0.47	NS	NS
4 h-post feeding	24.6	23.9	23.9	24.4	24.5	0.67	NS	NS
Mean	27.1	26.9	26.2	26.2	26.9	0.41	NS	NS
Butyrate (C_4_)								
0 h-post feeding	10.4	9.5	10.4	10	10.3	0.62	NS	NS
4 h-post feeding	11	11.9	12.6	12.5	12.5	0.40	NS	NS
Mean	10.7	10.7	11.5	11.3	11.4	0.32	NS	NS
C_2_:C_3_ ratio								
0 h-post feeding	2.0	2.0	2.1	2.2	2.1	0.12	NS	NS
4 h-post feeding	2.6	2.7	2.7	2.6	2.6	0.14	NS	NS
Mean	2.3	2.3	2.4	2.4	2.3	0.10	NS	NS

YFPKCP: yeast fermented palm cake kernel; SBM: soybean meal; SEM: standard error of the mean (*n* = 5); L: linear; Q: quadratic; NS: non-significant (*p* > 0.05).

**Table 6:** Diversity of rumen microorganisms in goats fed a concentrate diet comprising fermented palm kernel cake.

Attribute	YFPKCP Substitution SBM in Concentrate (%)						Contrast	
	0	25	50	75	100	SEM	L	Q
Total direct count								
Bacteria (×10^10^ cell/mL)								
0 h-post feeding	1.8 ^b^	2.6 ^ab^	3.0 ^a^	3.2 ^a^	3.5 ^a^	0.30	*	NS
4 h-post feeding	2.8 ^b^	3.2 ^b^	3.7 ^ab^	4.4 ^a^	4.5 ^a^	0.34	*	NS
Mean	2.3 ^b^	2.9 ^b^	3.4 ^ab^	3.8 ^a^	4.4 ^a^	0.38	*	NS
Total protozoa (×10^6^ cell/mL)								
0 h-post feeding	2.8	2.5	2.4	2.2	2.2	0.26	0.09	NS
4 h-post feeding	3.1	3.4	3.1	2.6	2.6	0.32	0.10	NS
Mean	3.0	2.9	2.7	2.4	2.3	0.26	0.07	NS
Fungal zoospores (×10^5^ cell/mL)								
0 h-post feeding	2.3 ^b^	2.5 ^b^	3.1 ^ab^	3.7 ^b^	3.9 ^b^	0.28	*	NS
4 h-post feeding	2.7 ^b^	2.5 ^b^	3.5 ^ab^	4.8 ^a^	4.9 ^a^	0.44	*	NS
Mean	2.5 ^b^	2.5 ^b^	3.3 ^ab^	4.3 ^a^	4.4 ^a^	0.36	*	NS

YFPKCP: yeast fermented palm cake kernel; SBM: soybean meal; SEM: standard error of the mean (*n* = 5); L: linear; Q: quadratic; NS: non-significant (*p* > 0.05); *: significantly different (*p* < 0.05); ^a, b^ Within rows not sharing a common superscript are significantly different (*p* < 0.05).

**Table 7:** Blood metabolized characteristics in goats fed fermented palm kernel cake diet.

Attribute	YFPKCP Substitution SBM in Concentrate (%)						Contrast	
	0	25	50	75	100	SEM	L	Q
Glucose, mg/dL								
0 h-post feeding	64.74	61.84	64.00	64.00	62.74	1.41	NS	NS
4 h-post feeding	64.60	66.00	66.00	67.72	65.80	1.06	NS	NS
Mean	64.67	63.92	65.00	65.00	64.27	1.07	NS	NS
Packed cell volume (PCV),%								
0 h-post feeding	28.60	26.80	27.80	28.80	28.70	0.72	NS	NS
4 h-post feeding	29.56	30.55	28.75	29.50	29.52	0.55	NS	NS
Mean	29.08	28.67	28.27	29.15	29.11	0.53	NS	NS
Blood urea nitrogen (BUN), mg/dL								
0 h-post feeding	20.35 ^a^	21.20 ^a^	18.41 ^b^	19.94 ^a^	20.22 ^a^	0.45	NS	*
4 h-post feeding	20.93	20.95	19.94	22.15	21.50	1.62	NS	NS

YFPKCP: yeast fermented palm cake kernel; SBM: soybean meal; SEM: standard error of the mean (*n* = 5); L: linear; Q: quadratic; NS: non-significant (*p* > 0.05); *: significantly different (*p* < 0.05); ^a, b^ Within rows not sharing a common superscript are significantly different (*p* < 0.05).

**Table 8:** Nitrogen consumption in goats given different levels of fermented palm kernel cake as a substitute for soybean meal.

Attribute	YFPKCP Substitution SBM in Concentrate (%)						Contrast	
	0	25	50	75	100	SEM	L	Q
Nitrogen (N) balance, g/d								
Total N intake	15.55	14.72	13.92	13.92	14.16	1.09	NS	NS
N excretion, g/d								
Fecal N	4.80 ^ab^	4.33 ^bc^	3.74 ^c^	4.15 ^bc^	5.50 ^a^	0.25	NS	**
Urinary N	2.01 ^a^	1.41 ^b^	1.49 ^b^	1.06 ^b^	1.33 ^b^	0.14	**	0.10
Absorbed N	10.70	10.38	10.18	9.80	8.66	0.96	NS	NS
Retained N	8.69	8.97	8.69	8.74	7.32	0.86	NS	NS
N output (% of N intake)								
Absorbed	68.51 ^a^	70.18 ^a^	72.72 ^a^	69.14 ^a^	61.02 ^b^	1.96	*	**
Retained	55.49 ^ab^	60.11 ^a^	62.08 ^a^	62.17 ^a^	51.59 ^b^	2.01	NS	**

YFPKCP: yeast fermented palm cake kernel; SBM: soybean meal; SEM: standard error of the mean (*n* = 5); L: linear; Q: quadratic; NS: non-significant (*p* > 0.05); *: significantly different (*p* < 0.05); **: *p* < 0.01; ^a, b, c^: Within rows not sharing a common superscript are significantly different (*p* < 0.05).

## Data Availability

Not applicable.

## References

[1] Hart S., Gipson T. (2019). Current situation and future prospects of the US goat industry. Prof. Agric. Work. J..

[2] Hartley C.W.S. (1988). The Oil Palm.

[3] Abdullah N., Hanita H., Kudo Y.H., Jalaludin S., Ivan M. (1995). The effects of bentonite on rumen protozoal population and rumen fluid characteristics of sheep fed palm kernel cake. Asian-Australas. J. Anim. Sci..

[4] Carvalho L.P.F., Melo D.S.P., Pereira C.R.M., Rodrigues M.A.M., Cabrita A.R.J., Fonseca A.J.M. (2005). Chemical composition, in vivo digestibility, N degradability and enzymatic intestinal digestibility of five protein supplements. Anim. Feed Sci. Technol..

[5] O’Mara F.P., Mulligan F.J., Cronin E.J., Rath M., Caffrey P.J. (1999). The nutritive value of palm kernel meal measured in vivo and using rumen fluid and enzymatic techniques. Livest. Prod. Sci..

[6] Punj M.L. (1981). Coordinated Research Project on Utilization of Agriculture by-Products and Industrial Waste Material for Evolving Economic Rations for Livestock.

[7] Mukendi G.M., Mitema A., Nelson K., Feto N.A. (2022). Bacillus Species of Ruminant Origin as a Major Potential Sources of Diverse Lipolytic Enzymes for Industrial and Therapeutic Applications. Bacilli Agrobiotechnology.

[8] Schlabitz C., Lehn D.N., de Souza C.F.V. (2021). A review of *Saccharomyces cerevisiae* and the applications of its byproducts in dairy cattle feed: Trends in the use of residual brewer’s yeast. J. Clean. Prod..

[9] Carpinelli N.A., Halfen J., Trevisi E., Chapman J.D., Sharman E.D., Anderson J.L., Osorio J.S. (2021). Effects of peripartal yeast culture supplementation on lactation performance, blood biomarkers, rumen fermentation, and rumen bacteria species in dairy cows. J. Dairy Sci..

[10] Brossard L., Chaucheyras-Durand F., Michalet-Doreau B., Martin C. (2006). Dose effect of live yeasts on rumen microbial communities and fermentations during butyric latent acidosis in sheep: New type of interaction. J. Anim. Sci..

[11] Acharya B.R., Pant S.R. (2021). Feeding regime evaluation for cost-effective milk production technology for crossbred cattle in Chitwan condition. Natl. Workshop Livest. Fish. Res. Nepal..

[12] Yuan K., Liang T., Muckey M.B., Mendonça L.G.D., Hulbert L.E., Elrod C.C., Bradford B.J. (2015). Yeast product supplementation modulated feeding behavior and metabolism in transition dairy cows. J. Dairy Sci..

[13] Zaworski E.M., Shriver-Munsch C.M., Fadden N.A., Sanchez W.K., Yoon I., Bobe G. (2014). Effects of feeding various dosages of Saccharomyces cerevisiae fermentation product in transition dairy cows. J. Dairy Sci..

[14] Guedes C.M., Goncalves D.M., Rodrigues A.M., Dias-da-Silva A. (2007). Effects of a *Saccharomyces cerevisiae* yeast on ruminal fermentation and fibre degradation of maize silages in cows. Anim. Feed Sci. Technol..

[15] Sheppy C., Bedford M.R., Partridge G.G. (2001). The current feed enzymes market and likely trends. Enzymes in Farm Animal Nutrition.

[16] Mullins C.R., Weber D., Block E., Smith J.F., Brouk M.J., Bradford B.J. (2013). Supplementing lysine and methionine in a lactation diet containing a high concentration of wet corn gluten feed did not alter milk protein yield. J. Dairy Sci..

[17] Hernández J., Benedito J.L., Abuelo A., Castillo C. (2014). Ruminal acidosis in feedlot: From aetiology to prevention. Sci. World J..

[18] Antai S.P. (1990). Enrichment of nutrient quality of cassava (*Manihot esculenta* Crantz) with microbial proteins. Plant Foods Hum. Nutr..

[19] Oboh G., Akindahunsi A.A. (2003). Biochemical changes in cassava products (flour & gari) subjected to *Saccharomyces cerevisiae* solid media fermentation. Food Chem..

[20] Schneider B.H., Flatt W.P. (1975). The Evaluation of Feed through Digestibility Experiment.

[21] Samuel M., Sagathewan S., Thomas J., Mathen G. (1997). An HPLC method for estimation of volatile fatty acids of ruminal fluid. Indian J. Anim. Sci..

[22] Crocker C.L. (1967). Rapid determination of urea nitrogen in serum or plasma without deproteinization. Am. J. Med..

[23] Association of Official Analytical Chemists (AOAC) (1998). Official Methods of Analysis.

[24] SAS (1990). SAS/STATTM User’s Guide (Release 6.03).

[25] Steel R.G.D., Torrie J.H. (1980). Principles and Procedures of Statistics: A Biometerial Approach.

[26] Jenkins T.C. (1993). Lipid metabolism in the rumen. J. Dairy Sci..

[27] NRC (2001). Nutrient Requirements of Dairy Cattle.

[28] Allen M.S. (2000). Effects of diet on short-term regulation of feed intake by lactating dairy cattle. J. Dairy Sci..

[29] Saxena S.K., Otterby D.E., Donker J.D., Good A.L. (1971). Effects of feeding alkali-treated oat straw supplemented with soybean meal or nonprotein nitrogen on growth of lambs and on certain blood and rumen liquor parameters. J. Anim. Sci..

[30] Huntington G.B., Archibeque S.L. (1999). Practical aspects of urea and ammonia metabolism in ruminants. Proc. Am. Soc. Anim. Sci..

[31] Hoover W.H. (1986). Chemical factors involved in ruminal fiber digestion. J. Dairy Sci..

[32] Satter L.D., Slyter L.L. (1974). Effect of ammonia concentration on rumen microbial protein production in vitro. Br. J. Nutr..

[33] Russell J.B., Rychlik J.L. (2001). Factors that alter rumen microbial ecology. Science.

[34] Cherdthong A., Wanapat M., Kongmun P., Pilajun R., Khejornsart P. (2010). Rumen fermentation, microbial protein synthesis and cellulolytic bacterial population of swamp buffaloes as affected by roughage to concentrate ratio. J. Anim. Vet. Adv..

[35] Anantasook N., Wanapat M., Cherdthong A., Gunun P. (2013). Changes of microbial population in the rumen of dairy steers as influenced by plant containing tannins and saponins and roughage to concentrate ratio. Asian-Australas. J. Anim. Sci..

[36] Polyorach S., Wanapat M., Cherdthong A., Kang S. (2016). Rumen microorganisms, methane production, and microbial protein synthesis affected by mangosteen peel powder supplement in lactating dairy cows. Trop. Anim. Health Prod..

[37] Forbes J.M., France J. (1993). Quantitative Aspects of Ruminant Digestion and Metabolism.

[38] Galbraith H., Miller T.B. (1973). Effect of metal cations and pH on the antibacterial activity and uptake of long chain fatty acids. J. Appl. Bacteriol..

[39] Abdullah N., Hutagalung R.I. (1988). Rumen fermentation, urease activity and performance of cattle given palm kernel cake based diet. Anim. Feed Sci. Technol..

[40] Preston R.L., Schnakanberg D.D., Pfander W.H. (1965). Protein utilization in ruminants. Blood urea nitrogen as affected by protein intake. J. Nutr..

[41] Kaneko J.J., Kaneko J.J. (1980). Appendixes. Clinical Biochemistry of Domestic Animals.

[42] Arcos-García J.L., Castrejón F.A., Mendoz G.D., Pérez-Gavilán E.P. (2000). Effect of two commercial yeast cultures with Saccharomyces cerevisiae on ruminal fermentation and digestion in sheep fed sugar cane tops. Livest. Prod. Sci..

[43] Firat A., Ozpinar A. (1996). The study of changes in some blood parameters (glucose, urea, bilirubin AST) during and after pregnancy in association with nutritional conditions and litter size in ewes. Turk. Vet. Anim. Sci..

[44] Ford E.J., Evans J., Robinson I. (1990). Cortisol in pregnancy toxemia of sheep. Br. Vet. J..

[45] Hove K., Halse K. Energy metabolism in ruminants with special reference on ketosis and fertility. Proceedings of the 5th International Conference on Production Disease in Farm Animals.

[46] Cherdthong A., Khonkhaeng B., Seankamsorn A., Supapong C., Wanapat M., Gunun N., Gunun P., Chanjula P., Polyorach S. (2018). Effects of feeding fresh cassava root with high-sulfur feed block on feed utilization, rumen fermentation and blood metabolites in Thai native cattle. Trop. Anim. Health Prod..

[47] Mahardika I.G., Sastradipradja D., Sutardi T., Sumadi I.K. (2000). Nutrient requirements of exercising Swamp buffalo, Bubalus bubalis II. Details of work energy of cows and its relation to heart rate. Asian-Australas. J. Anim. Sci..

[48] Rasedee A., Zainal J.A., Ragavan K., Halmi O. (1982). The effect of high and low protein diets on block parameters in lactating Friesian cow. Kajian Vet. Malays..

[49] Cherdthong A., Wanapat M. (2013). Manipulation of in vitro ruminal fermentation and digestibility by dried rumen digesta. Livest. Sci..

[50] Tamminga S. (1996). A review on environmental impacts of nutritional strategies in ruminants. J. Anim. Sci..

[51] Kang-Meznarich J.H., Broderick G.A. (1981). Effects of incremental urea supplementation on ruminal ammonia concentration and bacterial protein formation. J. Anim. Sci..

[52] Church D.C. (1979). Digestive Physiology and Nutrition of Ruminants.

[53] Gunun P., Gunun N., Wanapat M., Cherdthong A., Polyorach S., Sirilaophaisan S., Wachirapakorn C., Kang S. (2018). In vitro rumen fermentation and methane production as affected by rambutan pee powder. J. Appl. Anim. Res..

[54] Anantasook N., Wanapat M., Cherdthong A., Gunun P. (2013). Effect of plants containing secondary compounds with palm oil on feed intake, digestibility, microbial protein synthesis and microbial population in dairy cows. Asian-Australas. J. Anim. Sci..

[55] Kang S., Wanapat M., Cherdthorng A. (2014). Effect of banana flower powder supplementation as a rumen buffer on rumen fermentation efficiency and nutrient digestibility in dairy steers fed a high-concentrate diet. Anim. Feed Sci. Technol..

[56] Cherdthong A., Wanapat M., Wongwungchun W., Yeekeng S., Niltho T., Rakwongrit D., Khota W., Khantharin S., Tangmutthapattharakun G., Phesatcha K. (2014). Effect of feeding feed blocks containing different levels of urea calcium sulphate mixture on feed intake, nutrients of digestibility and rumen fermentation in Thai native beef cattle fed on rice straw. Anim. Feed Sci. Technol..

